# Simultaneous Segmentation and Classification of Pressure Injury Image Data Using Mask-R-CNN

**DOI:** 10.1155/2023/3858997

**Published:** 2023-02-02

**Authors:** Mark Swerdlow, Ozgur Guler, Raphael Yaakov, David G. Armstrong

**Affiliations:** ^1^Department of Surgery, Keck School of Medicine of USC, Los Angeles, CA, USA; ^2^eKare, Inc., Fairfax, VA, USA

## Abstract

**Background:**

Pressure injuries (PIs) impose a substantial burden on patients, caregivers, and healthcare systems, affecting an estimated 3 million Americans and costing nearly $18 billion annually. Accurate pressure injury staging remains clinically challenging. Over the last decade, object detection and semantic segmentation have evolved quickly with new methods invented and new application areas emerging. Simultaneous object detection and segmentation paved the way to segment and classify anatomical structures. In this study, we utilize the Mask-R-CNN algorithm for segmentation and classification of stage 1-4 pressure injuries.

**Methods:**

Images from the eKare Inc. pressure injury wound data repository were segmented and classified manually by two study authors with medical training. The Mask-R-CNN model was implemented using the Keras deep learning and TensorFlow libraries with Python. We split 969 pressure injury images into training (87.5%) and validation (12.5%) subsets for Mask-R-CNN training.

**Results:**

We included 121 random pressure injury images in our test set. The Mask-R-CNN model showed overall classification accuracy of 92.6%, and the segmentation demonstrated 93.0% accuracy. Our F1 scores for stages 1-4 were 0.842, 0.947, 0.907, and 0.944, respectively. Our Dice coefficients for stages 1-4 were 0.92, 0.85, 0.93, and 0.91, respectively.

**Conclusions:**

Our Mask-R-CNN model provides levels of accuracy considerably greater than the average healthcare professional who works with pressure injury patients. This tool can be easily incorporated into the clinician's workflow to aid in the hospital setting.

## 1. Introduction

Pressure injuries (PIs) impose a substantial burden on patients, caregivers, and healthcare systems, affecting an estimated 3 million Americans and costing nearly $18 billion annually [1]. Worldwide, pressure injuries affect over 10 percent of hospitalized patients [2, 3]. Often, pressure injuries develop iatrogenically in older, complex patients and require care in long-term care facilities after hospital discharge [4, 5]. Although these wounds are prevalent in the elderly, including nursing home residents, they can affect any patient with prolonged tissue ischemia. Patients suffering from pressure injury may experience a decreased quality of life, pain, reduced mobility, disfigurement, and depression, and they are at an increased risk for developing life-threatening infections, malignancy, and recurrent wounds.

Pressure injuries occur when sustained pressure to an area of the body compromises blood supply leading to tissue damage and breakdown. These injuries range in severity from stage 1, nonblanchable erythema of intact skin, to stage 4, full-thickness skin and tissue loss with expose fascia, muscle, tendon, or bone. Accurate pressure injury staging remains clinically challenging; one study found that the nonexpert clinician correctly stages a pressure injury 23% to 58% of the time, and another study demonstrated low interrater reliability among nurses staging skin breakdown photographs with a Cohen's kappa of 0.33 [6, 7]. A caregiver with minimal or no formal medical training would likely stage a pressure injury with even less accuracy. Wound management technologies play an important role in chronic wound treatment, including pressure injury. New computer vision methods exist to aid in accurate wound healing monitoring [8] [9, 10]. However, most caregivers continue to use imprecise visual assessment, increasing the likelihood of inaccurate measurements, infection, prolonged treatment, and discomfort [11, 12].

Object detection and segmentation has been applied to new areas of application in recent years with the advance of new techniques. Simultaneous object detection and segmentation allows for the segmentation and classification of anatomical structures. Current image processing and machine learning techniques are sufficiently advanced to develop tools that can accurately stage pressure injury based on a smartphone or tablet image.

Prior efforts for pressure injury staging measurement, segmentation, and classification include the development of a convolutional neural network (CNN) that segments different tissue types, granulation, necrotic eschar, and slough, in pressure injury to determine wound severity [13]. Images required a preprocessing step and consisted only of stage III and IV pressure injuries. Another study uses a 3D CNN to first determine the region of interest of a pressure injury image and then segments tissues in that region for staging classification [14]. Again, only stage III and IV pressure injury images were used. Chakraborty et al. segmented wounds with a high degree of accuracy using a preprocessing technique followed by fuzzy K-means clustering [15]. Yee et al. used structure from motion algorithms to accurately reconstruct digital wounds from smartphone wound videos as compared to a 3D industrial camera [16]. Veredas et al. combined neural networks and Bayesian classifiers to segment an image's wound region, extract the region's color and texture features, and classify pressure injury severity [17].

In this study, we use the Mask-R-CNN algorithm, a deep learning algorithm from the region-based convolutional neural network (R-CNN) family, to segment and classify pressure injury stage 1-4 images [18]. Unlike previous studies which require multiple steps to stage a pressure injury—such as first segmenting the wound or classifying the wound into granulation, slough, and eschar—the proposed method simultaneously performs wound segmentation and staging. Mask-R-CNN is the newest addition to the R-CNN algorithms; Mask-R-CNN localizes, classifies, and segments in less time than its predecessors took to analyze images. Manually labeled pressure injury images were used to train the model. Algorithm performance is assessed by accurate detection of pressure injury stage and region. Manual feature extraction is not required for the proposed CNN model. Results from testing on images collected under a variety of conditions show that the proposed hybrid model of pressure injury staging and segmentation can feasibly integrate into and support daily clinic routine. This pressure injury Mask-R-CNN implementation is unique and has not been previously applied. The chief contributions of this study are as follows: (i) the application of Mask-R-CNN to determine if an image contains pressure injury and, if so, to segment the image to delineate its borders; (ii) the classification of the segmented area with a high level of accuracy; and (iii) the verification of more accurate results with this method than current classification by pressure injury caregivers.

## 2. Methods

The deep learning-based region detection with CNN feature (R-CNN) family is a common model for object detection and currently consists of R-CNN, Fast-R-CNN, Faster-R-CNN, and Mask-R-CNN [19, 20].

R-CNN starts with a selective search which suggests approximately 2000 regions of interest (RoI) [20]. These RoIs are demarcated by bounding boxes which are then analyzed by the CNN for various features. Then, class-specific linear support vector machines (SVMs) classify each RoI. This is a time-intensive process which requires high computing power because the ConvNet forward-pass is executed independently for each RoI [21].

For each image, Fast-R-CNN creates a convolutional feature map, generates a feature vector for each corresponding RoI, and inputs it into a fully connected (FC) layer with the softmax probability (classification) and real-value position (class bounding box) as outputs.

Faster-R-CNN employs region proposal networks (RPNs) as an attention mechanism that merges with Fast-R-CNN, utilizing shared computation [21, 22]. This dual structure combines a deep fully convolutional network (FCN) for region proposal with the Fast-R-CNN for detection. Each of these R-CNN iterations decreases the computation time.

Mask-R-CNN segments images using a new, additional third branch that applies fine pixel-to-pixel alignment [23]. An object mask is generated by altering the Faster-R-CNN's framework region of interest align (RoIAlign) layers that alter RoI pooling layers to overlay the extracted features on the input image [23–25]. Segmentation masks are created by inputting RoIs into a FCN [26]. Mask-R-CNN generates localization, segmentation, and classification tasks.

**Figure 1:**
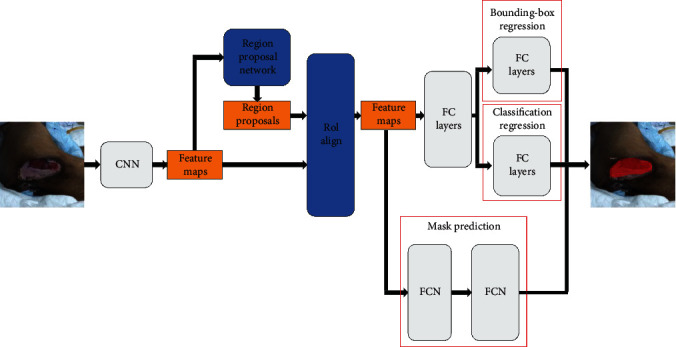
Framework of the proposed method.

In general, Mask-R-CNN's two-step process comprises a region proposal network and three RoIAlign-derived networks (Figure 1). Specifically, Mask-R-CNN uses a feature pyramid network (FPN) for the feature extraction process and ResNet101 to support the initial stage; it has four layers for convolution and one for deconvolution [23]. Its RoIAlign layer preserves the feature map size, and by avoiding quantization, it evades misalignment. Region proposals use preassigned anchors, and if there is an object inside the anchor, the model moves to each feature map pixel; this is the same approach as He et al. [25]. Once anchor coordinates are updated, bounding boxes are returned as object proposals. The bounding box loss (*L*_box_), classification loss (*L*_cls_), mask loss (*L*_mask_), and multitask loss (*L*) are the same as prior works [22–25, 27].

These features are input into three networks. Pressure injury masks are generated after undergoing four convolutions, one deconvolution, and ReLU filtering. Masks are downscaled to a size of 28×28. This allows for faster instance segmentation but may allow for the introduction of artifacts after scaling up. Then, the bounding box and classification branches are both sent through a common fully connected layer so that the model only classifies the bounding box interior.

**Figure 2:**
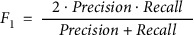
F1-score formula.

Images have one ground truth classification assigned during initial hand-labeling but can be predicted as one of the four possible classes. The performance metrics are defined as precision (fraction of predicted instances of a class that match their ground truth classification), recall (fraction of ground truth images of a class predicted as that class), accuracy (fraction of images correctly predicted as their ground truth class out of all images), and F1-score (see Figure 2).

**Figure 3:**
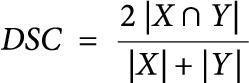
Dice-Sorensen coefficient formula.

Due to the binary nature of the classification, each pixel will be considered either “skin” indicating no wound or “pressure injury” for a wound. Each image pixel can be defined as true positive (wound pixel correctly classified as wound pixel), false positive (skin pixel incorrectly classified as wound pixel), true negative (skin pixel correctly classified as skin pixel), and false negative (wound pixel incorrectly classified as skin pixel). The performance metric used to quantify accuracy between predicted and ground truth segmentation is the Dice coefficient (Figure 3).

Our model starts with the pretrained weights for MS COCO and Matterport's code and utilizes the TensorFlow and Keras deep learning libraries with Python 3.6 [23, 28]. Training occurred for 50 epochs on an Intel(R) Xeon(R) CPU E5-2630 v3 at 2.40 GHz that has 128 GB memory and an NVIDIA Quadro K4200 that has a dedicated 4 GB and a shared 64 GB memory. This took roughly 24 hours. We used the same hyperparameters as those in the original Mask-R-CNN paper except we decreased our learning rate to 0.001 because the original value of 0.02 led to problematic increased TensorFlow weights [25].

### 2.1. Data Collection, Preprocessing, Simulation Environment, and Model Validation

The pressure injury wound data repository is provided by eKare Inc. (Fairfax, VA), which provides professional wound imaging and analysis services. Images are taken with a commercially available camera (eKare inSight) by users with no special training during the wound assessment process in clinic or their hospital stay. The pressure injury dataset, which includes stage 1, stage 2, stage 3, and stage 4 cases, was manually labeled for training and testing purposes. The applicability of the algorithm is improved by the variety of cases.

**Figure 4:**
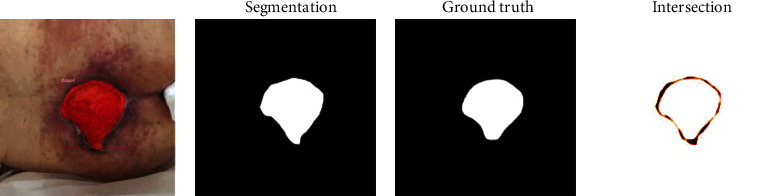
Pressure injury image with segmentation. (a) Pressure injury image with segmented area filled in red. (b) Segmented area shown in white. (c) Hand-labeled ground truth area in white. (d) Segmented area of disagreement corresponding to a Dice coefficient of 0.96.

**Figure 5:**
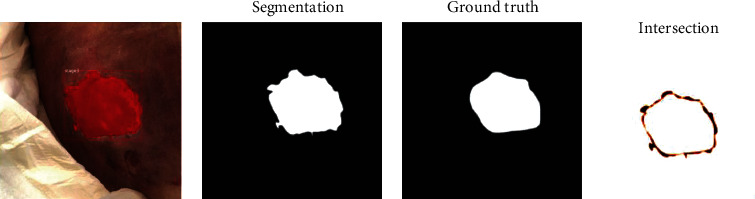
Pressure injury image with segmentation. (a) Pressure injury image with segmented area filled in red. (b) Segmented area shown in white. (c) Hand-labeled ground truth area in white. (d) Segmented area of disagreement corresponding to a Dice coefficient of 0.97.

Selected images are 1024 × 1024 pixels. Pressure injury images are hand-labeled at the pixel level where pixels in the wound region have a value of one; the rest of the pixel values in the image have a value of zero. Gauss (eKare Inc.) was used for image labeling. In total, 969 images contain pressure injury. Image examples are shown in Results (Figures 4 and 5). Areas detected and segmented as pressure injury are marked with red overlay.

Images were segmented and classified manually by two study authors with medical training; images that resulted in disagreements were discussed until a unifying conclusion was reached. Data augmentation via flipping was employed. The number of publicly available pressure injury images is limited and insufficient for the creation of a training dataset of deep learning-based wound border segmentation and tissue classification tasks. Further, finding pressure injury images with ground truths is either challenging or not possible. Moreover, image quality can be questionable. The Medetec wound database (Cardiff, UK) is a publicly available dataset that suffers degraded image quality because of the presence of mold growth on the original 35 mm transparencies. This further decreases the image resolution. In contrast, the unique eKare Inc. pressure injury image repository provides a sufficient number of high-quality images with ground truth data which enables high-quality training.

## 3. Results

We split 969 pressure injury images into training (87.5%) and validation (12.5%) subsets for Mask-R-CNN training. This proportion was chosen because the data-hungry Mask-R-CNN model requires a large number of images for adequate training. The bounding box of the region of interest, classification, and instance segmentation is trained simultaneously and predicted by the Mask-R-CNN networks.

### 3.1. Overall Accuracy

We included 121 random pressure injury images in our test set. Accuracy is the measurement of correctly classified instances. Based on our testing sample results, the Mask-R-CNN model showed overall classification accuracy of 92.6%, and the segmentation demonstrated 93.0% accuracy. We regulated model weights to minimize the loss function.

### 3.2. Classification

We used precision, recall, F1 score, and a confusion matrix as metrics to determine our accuracy. Precision is defined as the fraction of returned predicted instances of a class that correctly match their ground truth value of that class. Recall is defined as the fraction of ground truth images of a class that match their predicted returned value. The F1 score is the harmonic mean of the precision and recall and calculated according to the formula seen in Figure 2. The confusion matrix is a 4 × 4 matrix that represents the ground truth class as rows and the predicted class as columns; each value represents the number of images with a ground truth of that row returned as a predicted class of that column (e.g., 5 stage 4 images returned as predicted stage 3). Our results are listed in Tables 1 and 2.

### 3.3. Segmentation

Misclassification and segmentation accuracies are not correlated—incorrect classification does not imply segmentation error nor is the converse true. Additionally, in this hybrid approach, segmentation and classification form one output so they must be analyzed together. We continue the analysis of segmentation accuracy in instances with successful classification. We compare the predicted wound segmentation to the labeled ground truth segmentation using the Dice coefficient. We calculate the individual Dice indices according to the formula seen in Figure 3. A value of one represents perfect agreement between the predicted wound segmentation and ground truth segmentation, while a value of zero represents complete disagreement. The values of all Dice coefficients from a class were averaged into a single value to represent that class. Our Dice coefficients for stages one through four are 0.92, 0.85, 0.93, and 0.91, respectively. See Table 3.

Figures 4 and 5 show pressure injury segmentation and original images. The red region is predicted as pressure injury by the Mask-R-CNN. Overall, the model captures the wound boundaries with a high degree of accuracy.

## 4. Discussion

Pressure injuries affect millions of patients and cost our healthcare system billions of dollars each year [1]. Proper wound treatment depends on accurate identification of the injury stage. Prior studies have shown that accuracy among nonexpert clinicians treating pressure injuries is low. Additionally, the Centers for Medicare & Medicaid (CMS) does not provide reimbursement if the patient developed a pressure injury during the hospital stay [29]. Thus, prevention and accurate early detection of pressure injury represent an important challenge, especially in the hospital setting.

Our Mask-R-CNN provides levels of accuracy considerably greater than many healthcare professionals who work with patients with pressure injuries. Whereas the literature demonstrates the nonexpert clinician correctly staging pressure injuries 23%-58% of the time, the Mask-R-CNN has an overall accuracy of 92.6% [6, 7]. This tool will also be of great benefit to home caregivers who are untrained in pressure injury management. Given the ubiquity of smartphones and tablets and their increasing utilization in healthcare, this tool can easily be incorporated into the clinician's workflow to aid in the hospital setting.

There are important benefits of both our pressure injury detection method and image database. The Mask-R-CNN predicts both classification and segmentation simultaneously. Other CNNs either do only one or one after the other [13–17]. Our technique achieves high accuracy with decreased computation time.

We trained our CNN with images from a database where all images are taken with the same camera from approximately the same distance (40-65 cm) from the wound. This gives our database a high quality of images because most wound databases do not meet either of these features. Further, this scheme represents a considerable improvement over collecting random images pulled off online search engines which would also not meet these criteria.

While our classification results did have some errors, only a single image was classified in a stage not adjacent to the ground truth. This was a ground truth stage 1 image, the least serious stage of pressure injury and easiest to determine for the average person. No stage 2 images were misclassified. Of the four stage 3 images that were misclassified, two were predicted as stage 2 and two were predicted as stage 4, and none were predicted as stage 1 images. Only two stage 4 images were misclassified and both as stage 3. Although we continue to work to eliminate error from our system, even with an error, the classification is always adjacent to the ground truth for stages 2-4. It will be especially important for our system to maintain a high degree of accuracy with stage 3 images as the difference between treatment for stage 2 and stage 4 injuries is substantial; however, the difference between a stage 2 and stage 4 injury should be somewhat obvious even from the untrained layperson [30].

A limitation of our Mask-R-CNN accuracy metric is the relatively few stage 1 pressure injury images included in our test set. Although important to detect early for prevention of further tissue damage, primary treatment for a stage 1 injury is to remove pressure from the area. Imaging may not be necessary, and this is likely why our dataset has a smaller proportion of this class. Importantly, for stage 2-4 injuries which require more intensive treatment and are more likely to be imaged, we demonstrate a high accuracy, as evidenced by F1 scores greater than 0.900.

Additionally, we did not include wounds smaller than 2×2 cm in our training or testing sets. Thus, we cannot speak to the Mask-R-CNN accuracy for small wounds. Lastly, our wounds are limited by anatomical distribution. Pressure injury can occur anywhere on the body where there is unrelieved pressure, especially in places where skin covers bony areas. Most commonly, this includes the sacrum, coccyx, hips, and heels but can also include elbows, knees, ankles, shoulder, and posterior skull. Our dataset skews largely to images of the sacrum and coccyx. Although we do not expect the anatomic wound location to play a large role in the results of our classifier, we are unable to confirm any changes in accuracy.

Traditionally, we would compare other CNNs on our image set and report a comparison of results. However, we are unaware of other CNNs that simultaneously predict classification and segmentation. Thus, we are unable to test our method against other methods for concurrent pressure injury classification and segmentation and report comparative results.

Future work includes continuing to eliminate error from our classification and segmentation models and making sure this tool is equitable for patients of all skin tones. Although our dataset has images of all skin tones, we do not have data to classify this. We will also work to add pressure injury images from more locations on the body.

## 5. Conclusion

We present an adaptation of the innovative classification and instance segmentation algorithm, Mask-R-CNN, applied to an image dataset consisting of pressure injury images in various stages. The model detects pressure injury in these images and delineates its boundaries using instance segmentation. The proposed model achieves 92.6% and 93.0% accuracy for pressure injury detection and segmentation tasks, respectively. The loss function graphs of the three encapsulated networks, classification, bounding box detection, and segmentation mask networks, display efficient training of the model.

Mask-R-CNN segments rescaled (smaller-28 × 28) versions of the original image (1024 × 1024). This makes the segmentation pixel resolution coarse. Reconfiguring the Mask-R-CNN internal network structure to analyze images similar in size to the original image would improve resolution. However, increased image resolution would require an even larger image dataset to cause the learning to converge because the number of parameters would increase exponentially. Although the current segmentation is coarse, it performs segmentation with a high degree of accuracy, as evidenced by our Dice coefficient scores. Thus, we believe that the tradeoff of performing segmentation on downsampled images is justified.

## Figures and Tables

**Table 1:** Accuracy metrics.

Accuracy metrics	Stage 1	Stage 2	Stage 3	Stage 4
Precision	1.000	0.900	0.919	0.944
Recall	0.727	1.000	0.895	0.944
F1 score	0.842	0.947	0.907	0.944

**Table 2:** Confusion matrix.

Confusion matrix		Predicted			
		Stage 1	Stage 2	Stage 3	Stage 4
Ground truth	Stage 1	8	2	1	0
	Stage 2	0	36	0	0
	Stage 3	0	2	34	2
	Stage 4	0	0	2	34

**Table 3:** Dice-Sorenson coefficients.

Dice-Sorenson coefficients	Dice coefficient
Stage 1	0.92
Stage 2	0.85
Stage 3	0.93
Stage 4	0.91

## Data Availability

The pressure injury wound data repository is provided by eKare Inc. Data is available on request.
